# Constraint‐based models for dominating protein interaction networks

**DOI:** 10.1049/syb2.12021

**Published:** 2021-05-28

**Authors:** Adel A. Alofairi, Emad Mabrouk, Ibrahim E. Elsemman

**Affiliations:** ^1^ Department of Computer Science and Information Technology, Faculty of Science Ibb University Ibb Yemen; ^2^ Department of Mathematics, Faculty of Science Assiut University Assiut Egypt; ^3^ College of Engineering and Technology American University of the Middle East Kuwait Kuwait; ^4^ Department of Information Systems, Faculty of Computers and Information Assiut University Assiut Egypt

## Abstract

The minimum dominating set (MDSet) comprises the smallest number of graph nodes, where other graph nodes are connected with at least one MDSet node. The MDSet has been successfully applied to extract proteins that control protein–protein interaction (PPI) networks and to reveal the correlation between structural analysis and biological functions. Although the PPI network contains many MDSets, the identification of multiple MDSets is an NP‐complete problem, and it is difficult to determine the best MDSets, enriched with biological functions. Therefore, the MDSet model needs to be further expanded and validated to find constrained solutions that differ from those generated by the traditional models. Moreover, by identifying the critical set of the network, the set of nodes common to all MDSets can be time‐consuming. Herein, the authors adopted the minimisation of metabolic adjustment (MOMA) algorithm to develop a new framework, called maximisation of interaction adjustment (MOIA). In MOIA, they provide three models; the first one generates two MDSets with a minimum number of shared proteins, the second model generates constrained multiple MDSets (k‐MDSets), and the third model generates user‐defined MDSets, containing the maximum number of essential genes and/or other important genes of the PPI network. In practice, these models significantly reduce the cost of finding the critical set and classifying the graph nodes. Herein, the authors termed the critical set as the k‐critical set, where k is the number of MDSets generated by the proposed model. Then, they defined a new set of proteins called the (k−1)‐critical set, where each node belongs to (k−1) MDSets. This set has been shown to be as important as the k‐critical set and contains many essential genes, transcription factors, and protein kinases as the k‐critical set. The (k−1)‐critical set can be used to extend the search for drug target proteins. Based on the performance of the MOIA models, the authors believe the proposed methods contribute to answering key questions about the MDSets of PPI networks, and their results and analysis can be extended to other network types.

## 1 INTRODUCTION

Protein–protein interaction (PPI) networks play a major role in understanding disease mechanisms [1]. In the last 20 years, many experimental methods have been developed to reveal the high‐quality structure of PPI networks in many organisms, such as humans and yeast [2, 3, 4, 5]. The exponential growth in biotechnology has led to the availability of a wide range of databases describing PPI networks [6, 7]. Therefore, system‐level representation of the PPI network provides an opportunity to select a subset of genes that play an important role in cell viability, such as essential genes and cancer target genes [8].

In graph theory, the minimum dominating set (MDSet) is the smallest subset in which every other node in the network must be connected to at least one node of the MDSet [8, 9, 10]. The MDSet has been successfully applied in biological networks to reveal the correlation between structural analysis and biological function [8, 9, 11, 12, 13, 14, 15, 16, 17, 18, 19, 20]. For example, Wuchty [8] and Wakai et al. [16] applied an MDSet model and found an MDSet enriched with essential, cancer‐related, disease genes, and identified drug‐target proteins. Their model can identify only one MDSet, although the PPI networks contained many MDSets [8, 13, 17]. The critical set that contains common nodes in all MDSets of the PPI network has important locations in the PPI network and can be enriched with biological functions [12, 20]. Interactions with the critical nodes have eminent effects on the targeted network topology [12]. Therefore, discovering and testing the featured MDSets, and efficiently identifying the critical nodes are important for the analysis of PPI networks, as well as for ensuring model robustness [11].

Determining the MDSet is an NP‐complete problem [10], but no algorithm can find the MDSet in polynomial time [21]. Nacher and Akutsu [22] suggested an integer linear programming representation (ILP‐based) model to determine an optimal solution for the MDSet problem. Wuchty [8] applied the ILP‐based model to human and yeast PPI networks. Zhang et al. [13] developed the centrality‐corrected MDSet model that considers the degree and the betweenness centralities of proteins. Their model subsequently found more functionally significant proteins in essential genes, disease‐associated genes, ageing genes, and virus‐targeted genes. Despite their results, they concluded that relying on topological properties is not enough to predict the important proteins for consideration [17]. In this work, the authors hypothesised that the significance of enrichment analysis is affected by the algorithm used to determine the MDSet [23, 24], as deciding on the best MDSet for dominating the whole network is difficult [13]. Grinstead and Slater [25] reported that finding two or more MDSets with minimum intersection is an NP‐hard problem. Moreover, the set of shared nodes among all MDSets of the PPI network is called the critical set [20]. Wuchty et al. [12] found that in PPI networks, the critical set of proteins plays an important role in phosphorylation and regulatory events in their interactions.

Herein, a new framework is introduced, called maximisation of interaction adjustment (MOIA), to generate multiple MDSets for a given PPI network. The proposed MOIA is adopted from the minimisation of metabolic adjustment (MOMA) and linear MOMA algorithms used in metabolic networks [27, 28]. In MOIA, the authors developed a new model that generates two MDSets with the maximum differences between their nodes. The shared nodes between these two MDSets can be seen as the essential nodes that tightly contain the critical set of this network. Therefore, by calling on the optimisation algorithm only once, the proposed model encloses the critical set by defining the intersection between the generated MDSets. Then, the developed model was further extended to generate k‐MDSets with large differences between all of them, where k is the number of MDSets. Using these k‐MDSets, all nodes in the PPI network can be classified and the critical set precisely defined, named here as the k‐critical set. In addition, a new set of proteins appearing in (k−1)‐MDSets was extracted and this set was identified as the (k−1)‐critical set. Experimentally, it was found that the (k−1)‐critical set is equally as important as the k‐critical set and can be used to extend the search process for drug target proteins. Finally, an additional model was introduced to identify a specified MDSet when the user selects certain nodes as the dominating nodes. The authors believe that the MOIA method could be used to analyse biological and other networks to find the multiple and user‐defined constrained minimum dominating set. This approach can also contribute to ranking the nodes in the considered data network.

## 2 DOMINATING PROTEIN INTERACTION NETWORKS

### 2.1 Basic model of the MDSet problem

**FIGURE 1:**
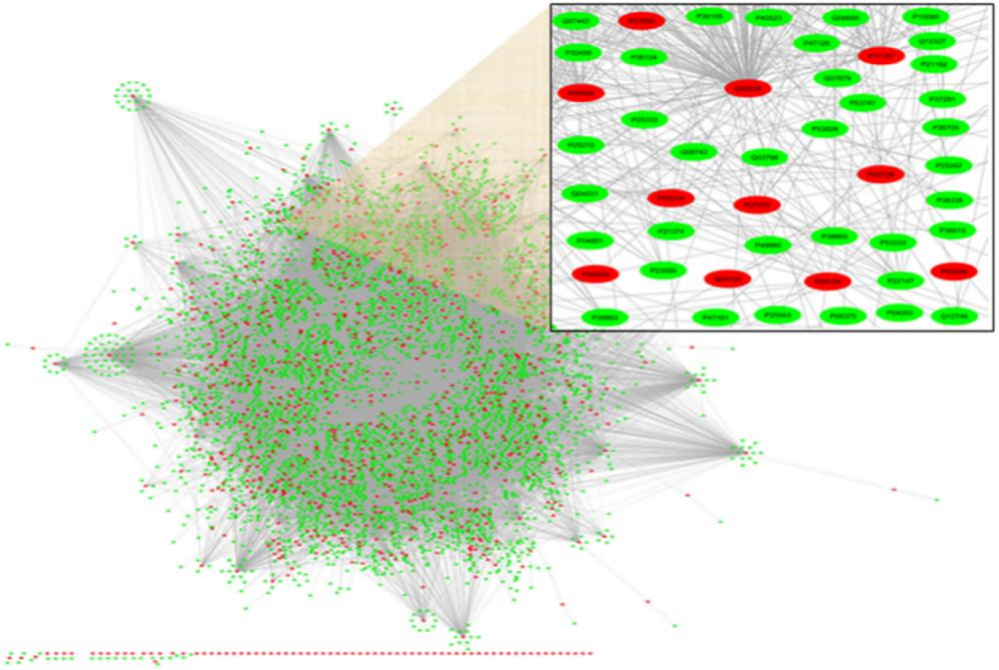
The yeast protein–protein interaction (PPI) network, where the set of red nodes represents an minimum dominating set (MDSet). Cytoscape tool [26] was used to draw this figure

The PPI network shown in Figure 1, drawn with the Cytoscape tool [26], could be described as an undirected graph G(V,E) where proteins are represented as the nodes V of the graph and the interactions between these proteins are represented as the edges E of the consideration graph. The adjacency matrix A(n×n) can be used to represent this graph, where n is the number of proteins in the PPI network, $A_{ij}=1$ if the protein i interacts with the protein j or i=j, and$\;A_{ij}=0,$ otherwise. A set D⊂V of proteins is considered as a dominating set if every node in V is either an element of D or adjacent to an element of D. The minimum dominating set of V is the smallest dominating set for the given network [8, 13].

**FIGURE 2:**
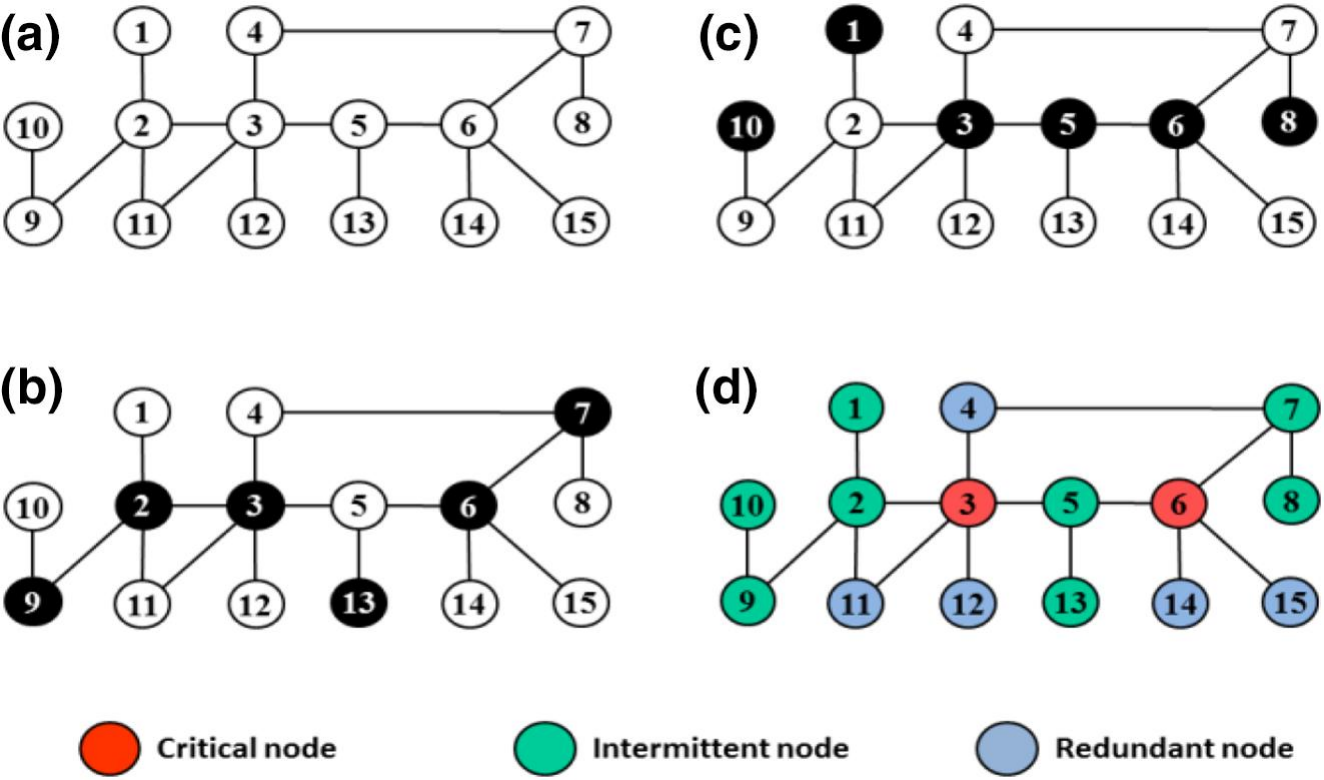
Dominating sets of a network and classifications of its nodes. (a) The original graph. (b, c) Two minimum dominating set (MDSets) of the graph. (d) Explanation of the node types in terms of critical, intermittent, and redundant nodes

Nacher and Akutsu [20] classified the nodes of the considered graph, based on being in the generated MDSets, into three types: critical nodes (belong to every MDSet), intermittent nodes (may belong to one MDSet), and redundant nodes (never belong to any MDSet). For example, Figure 2 shows a toy graph where each node has its category. This graph contains more than 10 MDSets, two of which are shown in Figures 2(b, c). In Figure 2(d), the red node‐set {3,6} represents the critical set of the graph, the green node‐set {1,2,5,7,8,9,10,13} is the intermittent set of the graph, and the remaining blue node‐set {4,11,12,14,15} forms the redundant set of the graph. Mathematically, the MDSet problem of PPI networks can be formulated as a binary integer‐programming problem as:

(1) $$\begin{array}{l} Objective\;:min\;\overset{n}{\underset{j=1}{\sum }}x_{j}\\ \;Subject\;to.\;\sum_{j=1}^{n}x_{j}\geq 1,\\ \;x_{i}\in \left\{0,1\right\},\;i=1,2,\ldots ,n \end{array}$$

The last constraint, $x_{j}\in \left\{0,1\right\}$ can be replaced by the relaxation constraint$\;0\leq x_{i}\leq 1,\;i=1,2,\ldots ,n$. The resulting integer‐programming problem can be solved using a branch‐and‐bound algorithm [29] or the simplex algorithm [30]. The ILP solvers can be used to solve the model in Equation (1). Herein, the authors use the MOSEK library (MOSEK ApS, Copenhagen, Denmark) under the MATLAB programming environment (Mathwork Inc.) as the main solver for the ILP problems [31]. MOSEK solver uses the interior point method along with the branch‐and‐bound algorithm [32, 33, 34] as a default algorithm for the resulting integer optimisation problem. Several MOSEK subroutines are used to solve ILP problems in the form:

$$\begin{array}{l} Objective\;:min\;C^{T}x\\ Subject\;to.\\ \;L^{c}\leq Ax\leq U^{c},\\ \;L^{x}\leq x\leq U^{x,} \end{array}$$

where A represents the adjacency matrix of the PPI network with n nodes. Moreover, x represents the solution vector, and $C,L^{c},U^{c},L^{x}$ and $U^{x}$ can be defined based on the proposed model. Therefore, to solve the ILP problem described in model (1) a solver subroutine is created that receives the adjacency matrix $A_{n\times n}$ and the remaining vectors; $C,L^{c},U^{c},L^{x}$ and $U^{x}$. Then, the MOSEK subroutine “MOSEKOPT” [33] is used to solve the ILP problem and return the MDSet M as the following:

$$M=Solver\left(C,A,L^{c},U^{c},L^{x},U^{x}\right),$$

The output of Solver is a binary vector (0–1 elements) of length n, where the set of elements of values 1 forms the resulting MDSet.

### 2.2 Multiple MDSets of PPI networks

Several MDSets can be found for a given PPI network, and each ILP solver can return a different solution according to its algorithm [11, 35]. Despite the presence of many MDSets in the PPI network, finding them all and defining their constraints is very difficult [13]. Consequently, finding two or more of these MDSets with a minimum intersection is an NP‐hard problem [25]. In addition, extracting important and critical proteins from PPI networks and classifying their nodes is another challenging and time‐consuming problem [12, 13, 15, 35]. Herein, the authors developed new MDSet models that can be used to:

1. Reduce the computational cost used in finding the critical, intermittent, and redundant sets, whereas the traditional methods find these sets after calling on the solvers n times, where n is the size of the PPI network.
2. Find new sets of proteins that have different criticalness degrees.
3. Allow the user to find a special MDSet that contains the maximum number of user‐defined proteins.
4. Validate the concept of the MDSet being enriched with the essential genes and biological functional categories.

## 3 PROPOSED METHODS

The MOMA and linear MOMA algorithms [27, 28] were adopted in metabolic networks to extend the ILP model in Equation (1) to generate several MDSets for PPI networks, where one or more of these MDSets may have biological functions. As the MOMA algorithms are famous algorithms in the constraint‐based reconstruction and analysis (COBRA) of metabolic models [36], the authors called their developed models constrained‐based models for dominating PPI networks (https://github.com/Alofairi1976/MOIA). Mainly, the aim was to generate MDSets with the largest number of differences among them. These different MDSets can be used to identify critical nodes, which reflect the effective proteins and gain important information about the PPI network. For example, the network in Figure 2 has 10 MDSets, however, only two of these MDSets shown in Figures 2(b, c) are sufficient to find the critical set as shown with the red in Figure 2(d).

**FIGURE 3:**
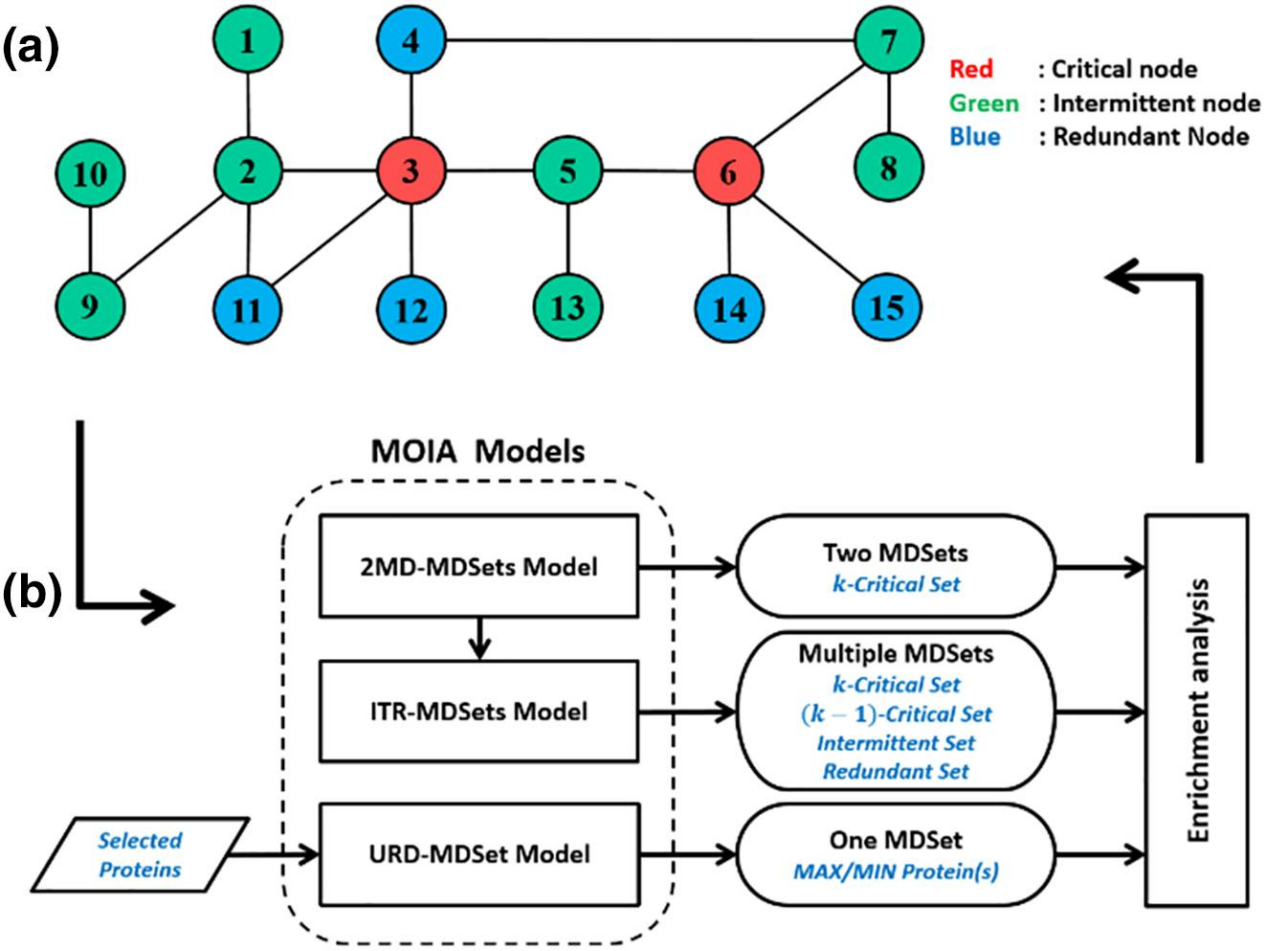
The structure of the proposed MOIA method. (a) A simple graph illustrates the types of minimum dominating set (MDSet) nodes or proteins. (b) MOIA pipeline that describes the developed models along with model's results k‐critical and (k–1)‐critical sets maximiszation of interaction adjustment (MOIA)

The proposed method comprises three main stages, as shown in Figure 3. The first stage refines the given data set using suitable data preprocessing techniques, which involve PPI data collection, protein selection, and graph implementation (the adjacency matrix). The second stage involves employing one model picked from three developed models: The two most different MDSets (2MD‐MDSets) model are the iterative MDSets (ITR‐MDSets) model, and the user‐defined MDSet (URD‐MDSet) model. The 2MD‐MDSets model aims to generate two MDSets simultaneously with the maximum number of different nodes between these MDSets. The ITR‐MDSets model can be used to generate many different MDSets. The URD‐MDSet model can generate an MDSet containing specific nodes which are determined by the user. In the third stage of the proposed MOIA method, the obtained results are discussed and interpreted. These results include several MDSets generated under different criteria to be used for determining the k‐critical, the intermittent, and the redundant set proteins. In this research, the authors highlight the importance of what they call the (k−1)‐critical set in the PPI network. In the following subsection, the Basic‐MDSet model in [8] is discussed. Then, the proposed models are introduced in the remaining subsections. To express the algorithms proposed herein, the following notations are defined:

- $I_{n\times n}$: the n‐by‐n identity matrix with ones on the main diagonal and zeros elsewhere.
- $J_{n\times m}$: the matrix of ones, where all n‐by‐m entries are ones.
- $O_{n\times m}$: the matrix of zeros, where all n‐by‐m entries are zeros.
- $A_{n\times n}$: the adjacency matrix, where all n‐by‐m entries are binaries; 0 or 1.
- X\Y: the set of all elements belongs to vector X but not vector Y.
- X∪Y: the union of vectors X and Y.
- |X|: the size, number of ones, of the vector X.

### 3.1 ILP‐based Basic‐MDSet model

Wuchty [8] applied the ILP‐based model [22] to find an optimal solution for the MDSet problem of PPI networks as follows: the solution of the problem in Equation (1) is a binary vectorx, where $x_{i}=1$ if protein i belongs to the generated MDSet and$\;x_{i}=0$ otherwise. Algorithm 1 introduces a pseudo code that describes the steps used to translate the implementation of the proposed model in Equation (1).

Algorithm 1 **Basic‐MDSet model**

1

1. Initialisation:

1.1. Read the number of nodes n and the data file.

1.2.  Create the adjacency matrix $A_{n\times n}.$

2. Build the model as described in Equation (1):

2.1. Set $C=J_{1\times n}$

2.2. Set $L^{c}=J_{1\times n}$ and $U^{c}=nJ_{1\times n}.\;$

2.3. Set $L^{x}=O_{1\times n}$ and $U^{x}=J_{1\times n}.$

3. Compute the MDSet $M=Solver\left(C,A,L^{c},U^{c},L^{x},U^{x}\right).$

4. Return the MDSet M

### 3.2 2MD‐MDSets model

Segre et al. [27] introduced the MOMA method that minimises flux distributions between mutant and wild‐type fluxes. Moreover, Zhang et al. [13] reduced the difference (i.e., to increase the overlap) between the generated MDSets using different optimisation solvers. In contrast, the MOMA method is adopted here to design an ILP‐model that can generate two MDSets simultaneously with the maximum number of different nodes between them. Specifically, two variables, x and y, can be used to represent two MDSets in the new system as follows:

(2) $$\begin{array}{l} Objective\;:max\overset{n}{\underset{j=1}{\sum }}│x_{j}-y_{j}│\\ Subject\;to.\\ \;\overset{n}{\underset{j=1}{\sum }}A_{ij}x_{j}\geq 1,\;i=1,2,...n.\\ \;\overset{n}{\underset{j=1}{\sum }}A_{ij}y_{j}\geq 1,\;i=1,2,...n.\\ \;\overset{n}{\underset{j=1}{\sum }}x_{j}=│MDSet│\\ \;\;\overset{n}{\underset{j=1}{\sum }}y_{j}=│MDSet│\\ \;x_{i}\in 0,1\;i=1,2,\ldots ,n,\\ \;y_{i}\in \left\{0,1\right\}\;i=1,2,\ldots ,n, \end{array}$$

where n represents the number of nodes or proteins in the targeted network and |MDSet| is the size of the generated MDSet using the model in Equation (1).

The intersection between the two MDSets x and y may represent the critical nodes in the graph. Therefore, the proposed model can quickly produce the critical set compared to the traditional method [37]. To adjust the model for linear programming techniques, $z_{i}$ is used as a new binary variable that satisfies the following two constraints:

$$\;x_{i}+y_{i}+z_{i}\;\leq 2,\;and\;0\leq x_{i}+y_{i}-z_{i}.$$

It is clear that $z_{i}=1$ is the best value if $x_{i}$ and $y_{i}$ are different, and $z_{i}$  = 0 is the best value if $x_{i}$ and $y_{i}$ are similar. Equation (3) describes the final developed model to generate the two most different MDSets by a suitable ILP solver:

(3) $$\begin{array}{l} Objective:max\overset{n}{\underset{j=1}{\sum }}z_{j}\\ Subject\;to:\;\\ \;\overset{n}{\underset{j=1}{\sum }}A_{\mathrm{ij}}x_{j}\geq 1,\;i=1,2,...n.\\ \;\overset{n}{\underset{j=1}{\sum }}A_{\mathrm{ij}}y_{j}\geq 1,\;i=1,2,...n.\\ \;\overset{n}{\underset{j=1}{\sum }}x_{j}=│\text{M}\text{D}\text{S}\text{e}\text{t}│\\ \;\overset{n}{\underset{j=1}{\sum }}y_{j}=│\text{M}\text{D}\text{S}\text{e}\text{t}│\\ \;x_{i}+y_{i}+z_{i}\leq 2\;i=1,2,\ldots ,n,\\ \;x_{i}+y_{i}-z_{i}\geq 0\;i=1,2,\ldots ,n,\\ \;x_{i}\in \left\{0,1\right\}\;i=1,2,\ldots ,n,\\ \;y_{i}\in \left\{0,1\right\}\;i=1,2,\ldots ,n,\\ \;z_{i}\in \left\{0,1\right\}\;i=1,2,\ldots ,n. \end{array}$$

Algorithm 2 introduces a pseudo code that describes the steps used to translate the proposed model in Equation (3).

Algorithm 2 **2MD‐MDSets model**

1

1. Initialisation:

1.1. Read the number of nodes n and the data file.

1.2.  Create the adjacency matrix $A_{n\times n}.$

2. Call Algorithm 1 to find the MDSet M and compute its size |M|.

3. Build the model as described in Equation (3):

3.1. Set $BigC=\left[\begin{array}{lll} O_{1\times n} & O_{1\times n} & J_{1\times n} \end{array}\right]$

3.2. Set $BigA=\left[\begin{array}{l} \begin{array}{lll} A_{n\times n} & O_{n\times n} & O_{n\times n}\\ O_{n\times n} & A_{n\times n} & O_{n\times n}\\ J_{1\times n} & O_{1\times n} & O_{1\times n} \end{array}\\ \begin{array}{lll} O_{1\times n} & J_{1\times n} & O_{1\times n}\\ I_{n\times n} & I_{n\times n} & I_{n\times n}\\ I_{n\times n} & I_{n\times n} & -I_{n\times n} \end{array} \end{array}\right]$

3.3. Set $BigL^{c}=\left[\begin{array}{l} \begin{array}{l} J_{n\times 1}\\ J_{n\times 1}\\ \left|M\right| \end{array}\\ \begin{array}{l} \left|M\right|\\ O_{n\times 1}\\ O_{n\times 1} \end{array} \end{array}\right]$ and $BigU^{c}=\left[\begin{array}{l} \begin{array}{l} nJ_{n\times 1}\\ nJ_{n\times 1}\\ \left|M\right| \end{array}\\ \begin{array}{l} \left|M\right|\\ 2J_{n\times 1}\\ 2J_{n\times 1} \end{array} \end{array}\right]$

3.4. Set $BigL^{x}=O_{3n\times 1}$ and $BigU^{x}=J_{3n\times 1}$

4. Compute the set $BigM=Solver\left(BigC,BigA,BigL^{c},BigU^{c},BigL^{x},BigU^{x}\right).$

5. Extract the MDSets $M_{1}$ and $M_{2}$ from BigM, where $BigM=\left[M_{1}\;M_{2}\;O_{1\times n}\right].$

6. Return the two MDSets $M_{1}\;\text{and}\;M_{2}.$

### 3.3 ITR‐MDSets model

The proposed model, then, was further extended to generate multiple MDSets that cover all intermittent nodes in the PPI networks. The variable x in Equation (3) was treated as an input vector of binary values in which $x_{i}=1\;$if the node i belongs to any resultant MDSet and $x_{i}=0\;$otherwise. The obtained model can be expressed as in Equation (4). The implementation of the model can be iterated to generate a new MDSet. The value of x is updated in every iteration. This loop is stopped when there is no change in the vectorx. As a result, the algorithm generates multiple MDSets with maximum differences between all of them.

(4) $$\begin{array}{l} \text{Objective}\;:\;\text{max}\;\overset{n}{\underset{j=1}{\sum }}z_{j}\\ \text{Subject}\;\text{to}.\;\\ \;\overset{n}{\underset{j=1}{\sum }}A_{ij}y_{j}\geq 1,\;i=1,2,\ldots n\\ \;\overset{n}{\underset{j=1}{\sum }}y_{j}=│\text{MDSet}│\\ \;y_{i}+z_{i}\leq 2-x_{i},\;i=1,2,...n.\\ \;y_{i}-z_{i}\geq -x_{i},\;i=1,2,...n.\\ \;x_{i}\in 0,1\;i=1,2,...n.\\ \;y_{i}\in 0,1\;i=1,2,...n.\\ \;z_{i}\in 0,1\;i=1,2,...n. \end{array}$$

Algorithm 3 introduces a pseudo code that describes the steps using to translate the proposed model in Equation (4).

Algorithm 3 **ITR‐MDSets model**

1

1. Initialisation:

1.1. Read the number of nodes n and the data file.

1.2.  Create the adjacency matrix $A_{n\times n}.$

2. Use Algorithm 2 to find two MDSets $M_{1}$ and $M_{2}.$

3. Set $X=O_{1\times n},\;X_{new}=M_{1}\cup M_{2}\;$and set the counter l=3.

4. While $\left|X_{new}\right|>0,$ repeat Steps 4.1–4.8

4.1. Set $X=X\cup X_{new}.$

4.2. Build the model as described in Equation (4):

4.3. Set $BigC=\left[\begin{array}{ll} O_{1\times n} & J_{1\times n} \end{array}\right]$

4.4. Set $BigA=\left[\begin{array}{l} \begin{array}{ll} A_{n\times n} & O_{n\times n}\\ J_{1\times n} & O_{1\times n}\\ I_{n\times n} & I_{n\times n} \end{array}\\ \begin{array}{ll} I_{n\times n} & -I_{n\times n} \end{array} \end{array}\right]$

4.5. Set $BigL^{c}=\left[\begin{array}{l} \begin{array}{l} J_{n\times 1}\\ \left|M_{1}\right|\\ O_{n\times 1} \end{array}\\ -X^{T} \end{array}\right]$ and $BigU^{c}=\left[\begin{array}{l} \begin{array}{l} nJ_{n\times 1}\\ \left|M_{1}\right|\\ 2-X^{T} \end{array}\\ J_{n\times 1} \end{array}\right]$

4.6. Set $BigL^{x}=O_{2n\times 1}$ and $BigU^{x}=J_{2n\times 1}$

4.7. Compute the set $BigM=Solver\left(BigC,BigA,BigL^{c},BigU^{c},BigL^{x},BigU^{x}\right).$

4.8. Extract the MDSets $M_{1}$ from BigM, where $BigM=\left[O_{1\times n}\;M_{1}\right].$

4.9. Set $X_{new}=M_{l}\backslash X$ and l=l+1.

5. Return X and the multiple MDSets $M_{1},\;M_{2},\;M_{3},\;\ldots ,\;M_{k}.$

Here, after finding X and the multiple MDSets, $M_{1},\;M_{2},\;M_{3},\;\ldots ,\;M_{k},$ the following steps can be implemented to classify all proteins in the targeted PPI network:

1. The k‐critical set $=\underset{i}{\cap }M_{i},$ is the intersection of all MDSets $M_{1},\;M_{2},\;M_{3},\;\ldots ,\;M_{k}.$
2. The (k−1)‐critical set $=\underset{i}{\sum }\underset{j\neq i}{\cap }M_{j},$ is the set of all nodes that exist in (k−1) MDSets.
3. The intermittent set$=\underset{i}{\cup }M_{i},$ is the union of all MDSets $M_{1},\;M_{2},\;M_{3},\;\ldots ,\;M_{k}.$
4. The redundant set is the complement of the intermittent set.

### 3.4 URD‐MDSet model

Algorithm 4 describes the steps needed to generate the targeted MDSet by avoiding some specific nodes.

Algorithm 4 **URD‐MDSet model**

1

1. Initialisation:

1.1. Read the number of nodes n and the data file.

1.2. Create the adjacency matrix $A_{n\times n}.$

1.3. Read X; the vector of all nodes that will be avoided, if possible, in the targeted MDSet.

2. Use Algorithm 1 to find the MDSet M.

3. Build the model as described in Equation (4):

3.1. Set $BigC=\left[\begin{array}{ll} O_{1\times n} & J_{1\times n} \end{array}\right]$

3.2. Set $BigA=\left[\begin{array}{l} \begin{array}{ll} A_{n\times n} & O_{n\times n}\\ J_{1\times n} & O_{1\times n}\\ I_{n\times n} & I_{n\times n} \end{array}\\ \begin{array}{ll} I_{n\times n} & -I_{n\times n} \end{array} \end{array}\right]$

3.3. Set $BigL^{c}=\left[\begin{array}{l} \begin{array}{l} J_{n\times 1}\\ \left|M\right|\\ O_{n\times 1} \end{array}\\ -X^{T} \end{array}\right]$ and $BigU^{c}=\left[\begin{array}{l} \begin{array}{l} nJ_{n\times 1}\\ \left|M\right|\\ 2-X^{T} \end{array}\\ J_{n\times 1} \end{array}\right]$

3.4. Set $BigL^{x}=O_{2n\times 1}$ and $BigU^{x}=J_{2n\times 1}$

4. Compute the set $BigM=Solver\left(BigC,BigA,BigL^{c},BigU^{c},BigL^{x},BigU^{x}\right).$

5. Extract the MDSets $M_{1}$ from BigM, where $BigM=\left[O_{1\times n}\;M_{1}\right].$

6. Return the MDSet $M_{1}.$

## 4 DATA SETS

In this section, a set of PPI networks used through numerical experiments to reflect the efficiency of the proposed models is presented. Six data sets are used from the High‐quality Interactomes (HINT) database version (3/10/2018), where these data sets have been collected from several interactome resources [38]. In addition, two data sets from the BioPlex (biophysical interactions of ORFeome‐based complexes) network [39] were used.

### 4.1 Human protein data sets

For human PPI networks, three different data sets obtained from *H. sapiens* in the HINT database (version 3/10/2018) [38] were considered. The first one of these data sets contains 63,684 high‐quality binary protein (HHQBP) interactions between 12,815 human proteins. The second data set contains 116,456 high‐quality co‐complex protein (HHQCP) interactions between 12,352 human proteins. However, a network of 180,140 combined protein (HCP) interactions between 15,744 human proteins is considered as the third data set.

### 4.2 Yeast protein data sets

Three different data sets of the yeast interacting [40] protein networks were considered. These data sets were obtained from *S*. *cerevisiae* in the HINT database (version 3/10/2018) [38]. The first data set under consideration contains 23,202 high‐quality binary protein (YHQBP) interactions between 5313 yeast proteins. The second data set contains 68,779 high‐quality co‐complex protein (YHQCP) interactions between 5246 yeast proteins. The last data set consists of 91,981 combined protein (YCP) interactions between 5959 yeast proteins.

### 4.3 Bioplex protein interaction network

Two versions of the protein interaction data set of the BioPlex network [39] were used. The first version, BIOPLEX1, had 23,744 proteins interactions between 7637 proteins, and the second, BIOPLEX2, had 56,553 protein interactions between 10,883 proteins. Moreover, these two data sets with 80,297 protein interactions between 11,540 proteins were also combined as (BIOPLEX12).

### 4.4 Liver proteins data set

The 28,553 protein interactions between 7148 liver tissue proteins (LTP) collected in [11] were used.

### 4.5 Enrichment analysis data sets

The following data sets for the biological functional enrichment analysis were used:

- Essential genes (EGs) data sets: 1110 yeast essential genes and 2032 human essential genes from the DEG database, which collects data about essential genes from the literature, were utilized [41].
- kinase genes (KGs) data sets: 538 human kinases reported by Cheng et al. [40] and yeast 127 kinases from the Yeast Kinase Interaction Database were used [42].
- Transaction factors (TFs) data sets: 1214 human transaction factors reported by Vaquerizas et al. [43] and 268 yeast transcription factors from the YeastTract database were used [44].
- Drug‐target genes (DGs) and pharmaceutics genes (PHGs) data sets: the DrugBank database was utilized to obtain 1214 and 568 genes for drug and pharmaceutics genes, respectively [45].
- Housekeeping genes (HKGs) data set: the Human Protein Atlas Database (available on the portal http://www.proteinatlas.org) was used to obtain 3804 housekeeping genes in the human network [9].

## 5 RESULTS AND DISCUSSION

In this section, the implementation and performance of the proposed algorithms for data sets under consideration are discussed. All numerical results were implemented on a system with an Intel (R) Core (MT) i5 processor of 2.53 GHz and 4.0 GB Ram.

### 5.1 Results of the Basic‐MDSet model

The Basic‐MDSet model in Equation (1) was applied on the human PPI networks from the HINT database version (3/10/2018) [38]; HHQBP, HHQCP, and HCP. Table 1 shows the results of this experiment compared with the results of Wuchty [8] for an old version of the HINT database. It was found that despite the current networks being larger than the previous networks by about 40%, the ratio of the MDSet's size (%MDSet) to the number of proteins in each network was less than 20%. In the HHQCP and HCP data sets, the ratio %MDSet was reduced to around 13% for the new version [35]. This result may be because of the increasing number of interactions. Table 2 shows the same results and analysis for the yeast, BioPlex and liver data sets, which was explained in Section 4. The results in Tables 1 and 2 indicate that the size of the MDSet is less than 20% of the number of proteins for all data sets, even with the increase in the number of proteins and interactions.

**TABLE 1:** Results of the Basic‐MDSet model compared with the results in Wuchty [8] for human PPI networks from the HINT database

	Hint human proteins wuchty [8]			Hint human proteins extended version [38]		
	HHQBP	HHQCP	HCP	HHQBP	HHQCP	HCP
No. of proteins	8073	3089	8,495	12,815	12,352	15,744
No. of protein interactions	24,306	6,768	28,627	63,684	116,456	180,140
|MDSet|	1517	704	1509	2398	1699	2081
%MDSet	**18.80%**	**22.80%**	**17.80%**	**18.70%**	**13.80%**	**13.20%**

Abbreviations: HCP, combined protein; HHQBP, high‐quality binary protein; HHQCP, high‐quality co‐complex protein; PPI, protein–protein interaction.

**TABLE 2:** Statistics of applying the Basic‐MDSet model for PPI networks under consideration; the HINT, BioPlex and Liver data sets

	Hint yeast proteins			Bioplex human proteins			Liver proteins
	YHQBP	YHQCP	YCP	BPLEX1	BPLEX2	BPLEX12	
No. of proteins	5313	5246	5959	7637	10,883	11,540	7148
No. of protein interactions	23,202	68,779	91,981	23,744	56,553	80,297	28,553
|MDSet|	921	287	431	1196	1727	1767	1250
%MDSet	**17.30%**	**5.50%**	**7.20%**	**15.66%**	**15.87%**	**15.31%**	**17.49%**

### 5.2 Importance of the 2MD‐MDSets model

**FIGURE 4:**
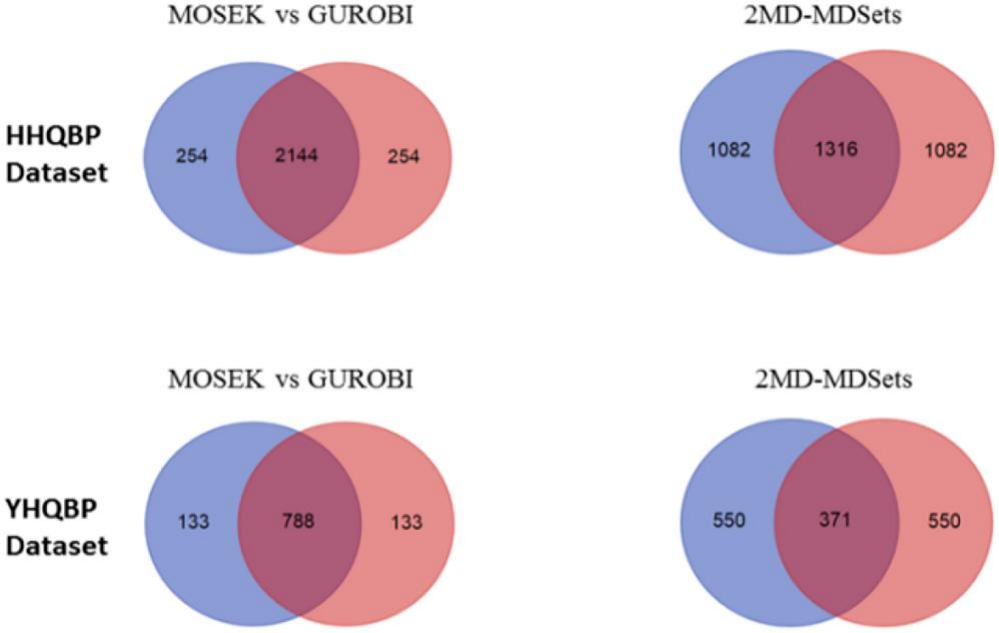
The overlaps between the MDSets generated by the MOSEK and GUROPI solvers compared with the overlaps between the MDSets generated by the 2MD‐MDSets model for the HHQBP and YHQBP data sets. The tool in [46] has been used to draw this figure

To show the efficiency of the proposed 2MD‐MDSets model in Equation (3), its results were compared with the Basic‐MDSet model in Equation (1) using two different solvers: MOSEK and GUROPI (Guropi Inc. Houston, TX, USA). Each solver generated one MDSet for the HHQBP data set, where the resulting MDSets intersected for 2,144 proteins. Similarly, two MDSets were generated using MOSEK and GUROPI solvers for the YHQBP data set, where these MDSets intersected for 788 proteins. Figure 4, drawn with the tool in [46], shows these results compared with the results of the 2MD‐MDSets model in Equation (3) for the same data sets. The number of overlapped proteins using the proposed model reduced from 2144 to 1316 in HHQBP data sets and from 788 to 371 in YHQBP data sets. The 2MD‐MDSets model was applied to several PPI networks, as given in Table 3. The proposed model could minimise the overlapping proteins, which may represent the critical set of each network. To validate the results of the 2MD‐MDSets model, the exact critical set of each network was evaluated with the traditional method [20], which can be concluded as follows:

1. Use the Basic‐MDSet model in Equation (1) to find the MDSet of the network and evaluate its size, m=|MDSet|.
2. Repeat the following steps for every $x_{v}\in \text{MDSet}.$
  2.1. Correct the model in Equation (1) by adding the new constraint $x_{v}=0.$
  2.2. Solve the new model to find a new MDSet.
  2.3. If |MDSet|>m, then add $x_{v}$ to the critical set.
3. Return with the critical set of proteins.

**TABLE 3:** Results of the 2MD‐MDSets model and the intersections between the resulting MDSets along with the critical set using the Basic‐MDSets model for each PPI network under study

	Hint human proteins			Hint yeast proteins				Bioplex human proteins			Liver proteins
	HHQBP	HHQCP	HCP	YHQBP	YHQCP		YCP	BPLEX1	BPLEX2	BPLEX12	
No. of proteins	12,815	12,352	15,744	5313	5246	5959		7637	10,883	11,540	7148
|MDSet|	2398	1699	2081	921	287	431		1196	1727	1767	1250
**2MD‐MDSets overlap**	**1316**	**689**	**982**	**371**	**91**	**158**		**553**	**731**	**716**	**479**
|CriticalSet|	**1315**	**670**	**968**	**364**	**90**	**154**		**547**	**716**	**709**	**476**
%Critical set	10.30%	5.40%	5.50%	6.90%	1.70%	2.60%		7.2%	6.58%	6%	6.66%

From Table 3, it can be concluded that the overlap of the resulting MDSets using the 2MD‐MDSets model in Algorithm 2, which was called on only once, is almost equal to the size of the critical set evaluated by calling on the Basic‐MDSet model hundreds/thousands of times for each data set. Moreover, it is expected that the extra proteins in the overlap between the resultant MDSets are important and may represent another important set in the PPI networks.

Ishitsuka et al. [47] used pre‐processing steps, before calling on the algorithm, to identify some of the critical nodes based on the topological structure of the PPI network. Identifying this set of nodes and marking it as critical nodes helps reduce the number of solver calls. Moreover, they stated that their algorithm reduces the computational time by about 180 times compared to the traditional method of finding the critical set of PPI networks. In Table 3, the basic model takes ∼100 seconds to find an MDSet of 2398 proteins in the HHQBP network. Therefore, traditional methods [20] call on the solver 2398 times to find the critical set, which equates to ∼2398×100=239,800 seconds. However, one call of Algorithm 2 with the 2MDSet model only takes ∼1020 seconds. Therefore, the proposed 2MDSet model determines the critical set up to be 235 times faster than the traditional methods, even without any pre‐processing steps.

### 5.3 Interpretation of ITR‐MDSets results

In this subsection, the focus is on the importance of the proposed ITR‐MDSets model and the interpretation of its output, specifically finding the critical, intermittent, and redundant sets of the PPI network very quickly compared to traditional methods discussed in the previous subsection. The ITR‐MDSets model starts by combining the two solutions, x and y, obtained from the 2MD‐MDSets model as x=x∪y. Then, the algorithm generates a new MDSet, y, using the model in Equation (4) where the differences between x (the input) and y (the output) are maximal. These two steps will be iterated until no new nodes could be added into x. Then, the algorithm returns k MDSets that will be used to find the critical, intermittent, and redundant sets according to steps explained in Section 3.

Table 4 summarises the results of the ITR‐MDSets model for the data sets under consideration. From Table 4, the critical set was evaluated very fast compared with the traditional method [20]. For example, the critical set of the HHQBP network is evaluated using only 13 iterations compared with 2398 iterations with the traditional method [20], as explained in the previous section.

**TABLE 4:** Results of the ITR‐MDSets model and evaluation of the critical, intermittent, and redundant sets for each data set

	Hint human proteins			Hint yeast proteins			Bioplex human proteins			Liver proteins
	HHQBP	HHQCP	HCP	YHQBP	YHQCP	YCP	BPLEX1	BPLEX2	BPLEX12	
No. of proteins	12,815	12,352	15,744	5313	5246	5959	7637	10,883	11,540	7148
|MDSet|	2398	1699	2081	921	287	431	1196	1727	1767	1250
**No. of MDSets**	**13**	**45**	**25**	**20**	**26**	**72**	**33**	**27**	**28**	**24**
|CriticalSet|	**1315**	**670**	**968**	**364**	**90**	**154**	**547**	**716**	**709**	**476**
|IntermittentSet|	2591	2779	2986	1413	616	868	5477	7555	8058	4792
|RedundantSet|	8909	8903	11,790	3536	4540	4937	2160	3328	3482	2356

### 5.4 Usage of the URD‐MDSet model

The URD‐MDSet model in Equation (4) is designed to generate MDSets with the maximum or minimum number of specific nodes selected by the user. For example, this model can be used to maximise/minimise the number of essential genes in the resulted MDSet. Li et al. [35] discussed the need for the computational models to predict the essential genes from the biological network. Wuchty [8] and Zhang et al. [13] used different techniques to evaluate the MDSet and concluded that their solutions were enriched with several essential genes. However, the number of essential genes in these MDSets is unpredictable and varies according to the algorithm used. In this experiment, the URD‐MDSet model will be used to increase the number of essential genes, and other important genes, in the resulting MDSets. Additionally, the proposed model can be used to answer the famous question "*Is each MDSet enriched with essential genes?*" In the literature, to answer this question, researchers used to randomly remove such proteins from the network and search for the MDSet for the modified network [8]. However, the proposed model can find the MDSet with the minimum number of essential genes in the network. Therefore, the proposed model can be used to answer this question efficiently and precisely. The URD‐Model was applied to find the MDSet with the minimum number of EGs in HHQBP and YHQBP PPI networks.

The authors obtained MDSets with 325 and 129 genes from the total EGs of 2032 and 1110 in HHQBP and YHQBP data sets, respectively. These MDSets are unenriched with EGs as will be explained in the next subsection. The cell needs all the essential genes [41, 48], kinase genes [40, 42], and transcription factor proteins [43] in signal transaction pathways. In this experiment, the URD‐MDSets model was constrained to maximise the number of EGs, KGs, TFs, DGs, and/or PHGs [45]. The results of the experiment are shown in Table 5. These results prove that the generated MDSet can be constrained as desired, rather than maximising the number of nodes with specific features, such as degree number [13].

**TABLE 5:** Results of the URD‐MDSets model with different constraints to maximise important genes like EGs, KGs, TFs, DGs, and PHGs on the HHQBP PPI network

Constrained	No. of proteins						Time in seconds
	Dominating genes				Target genes		
	EGs.2058	KGs.464	TFs.1213	ALL.3191	DGs.1699	PHGs.568	
Max EGs	640	108	222	801	470	167	1095.53
Max kinase	504	142	209	705	479	162	1037.70
Max TFs	509	106	293	742	455	160	1015.49
Max drug	517	125	202	702	587	202	1012.78
Max pharm.	513	113	212	691	503	215	1045.32
Max kinase + TFs + EGs	617	132	270	859	478	162	1026.56
Basic model (Mosek)	501	107	210	675	469	165	103.15

Table 5 shows the time and cost to increase the essential genes in MDSet. This time consists of the execution time of the algorithm plus the time required to manually define the vector, x. The results showed that the proposed model significantly increased the number of EGs in the resulting MDSet compared to the number of EGs in MDSets generated by the basic model in Equation (1) using the Mosek solver. Nevertheless, the solution time for the URD‐MDSet model increased to 10 times the solution time for the basic model in Equation (1). These results are consistent with the trade‐off between the significance of the results and the computational cost [35].

### 5.5 Functional enrichment analysis

For the enrichment analysis for the resulting MDSets, Fisher exact test in R language was used [49]. In this test, the size of the PPI network and the size of the resulting MDSet were input along with the number of the important genes under consideration (ESs or KGs, etc.) and the number of these genes in the resulting MDSet. The output of the test is a p‐value, where p−value<0.05 means that the MDSet is enriched with the important genes.

Table 6 shows the results of the EGs' enrichment analysis for the first five MDSets generated by the ITR‐MDSets model for the HHQBP and YHQBP data sets. Moreover, for each data set, the URD‐MDSets is used to generate one MDSet with the minimum number of EGs and one MDSet with the maximum number of EGs. Table 7 shows the enrichment analysis for all MDSets, generated by the URD‐Model in Table 5. Although most of the MDSets are enriched with EGs, the PPI network may contain unenriched MDSets.

**TABLE 6:** Enrichment analysis using Fisher exact test of some MDSets generated for HHQBP and YHQBP PPI networks

MDSet	HHQBP data set					YHQBP data set				
	|MDSet|	No. EGs	p‐Value	EGs in the k‐critical set	EGs in the (k−1)‐critical set	|MDSet|	No. EGs	p‐Value	EGs in the k‐critical set	EGs in the (k−1)‐critical set
1	2398	458	5.7E‐06	240	95	921	185	2.5E‐01	93	35
2	2398	467	4.2E‐07	240	70	921	208	3.1E‐03	93	47
3	2398	524	6.6E‐17	240	165	921	215	3.9E‐04	93	81
4	2398	504	6.0E‐13	240	165	921	222	3.5E‐05	93	81
5	2398	508	1.1E‐13	240	165	921	217	2.1E‐04	93	80
Min EGs	2398	330	1.0 E+00	240	20	921	129	1.0 E+00	93	17
Max EGs	2398	640	1.9E‐50	240	165	921	279	2. 0E‐16	93	81

**TABLE 7:** Enrichment analysis using Fisher exact test of the generated MDSets of proteins among EGs, KGs, TFs, DGs, and PHGs on the HHQBP PPI network

Constrained	*р*‐value					
	Dominating genes				Target genes	
	EGs.2058	KGs.464	TFs. 1213	ALL.3191	DGs.1699	PHGs.568
Max EGs	1.90E‐50	7.12E‐03	6.62E‐01	1.29E‐25	1.47E‐22	1.79E‐10
Max kinase	5.97E‐13	2.19E‐10	9.25E‐01	1.44E‐08	5.51E‐25	3.87E‐09
Max TFs	6.89E‐14	1.32E‐02	4.16E‐07	5.52E‐14	8.60E‐19	1.24E‐08
Max drug	1.85E‐15	6.08E‐06	9.77E‐01	3.41E‐08	1.81E‐63	1.95E‐22
Max pharm.	1.16E‐14	1.25E‐03	8.85E‐01	6.70E‐07	4.69E‐32	5.34E‐28
Max kinase + TFs + EGs	2.13E‐42	1.31E‐07	6.17E‐04	1.39E‐40	1.04E‐24	3.87E‐09
Mosek solution	2.10E‐12	9.74E‐03	9.13E‐01	2.99E‐05	2.69E‐22	6.28E‐10

**FIGURE 5:**
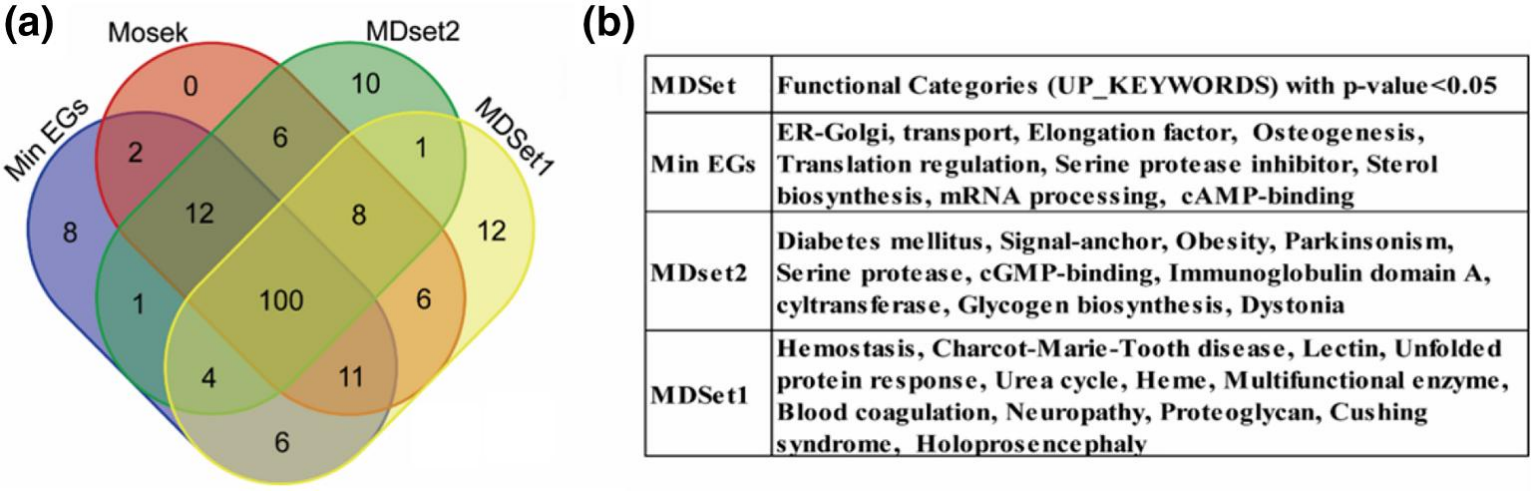
Comparison of shared biological functions categories (from DAVID tool [50]) for four MDSets: Mosek MDSet, Min EGs MDSet, MDSet1, and MDSet2 from the 2MD‐MDSets model. (a) The number of shared functional categories among these MDSets. (b) The unique functional categories in each MDSet with p−value<0.05

To verify that each MDSet has different biological functions, the DAVID tool [50] was used to annotate four MDSets: “Mosek”, the MDSet for the basic model, “Min EGS”, the MDSet with min number of essential genes (in Table 6), and “MDSet1” and “MDSet2” generated by the 2MD‐MDSets model (in Figure 4). Only functional categories with UP_KEYWORDS and p−value<0.05 (EASE score <0.05) were used. It was found that the majority of biological function categories are shared among these MDSets as in Figure 5(a). Additionally, three MDSets were found, each with some unique functional categories, in metabolism, RNA processing, translational regulations [Figure 5(b)]. For example, MDSet2 is enriched with diabetes mellitus with p−value<0.0063. Moreover, it was found that each set has different processes in metabolism, RNA processing, translation regulations.

### 5.6 Analysis of the (k−1)‐critical set

**FIGURE 6:**
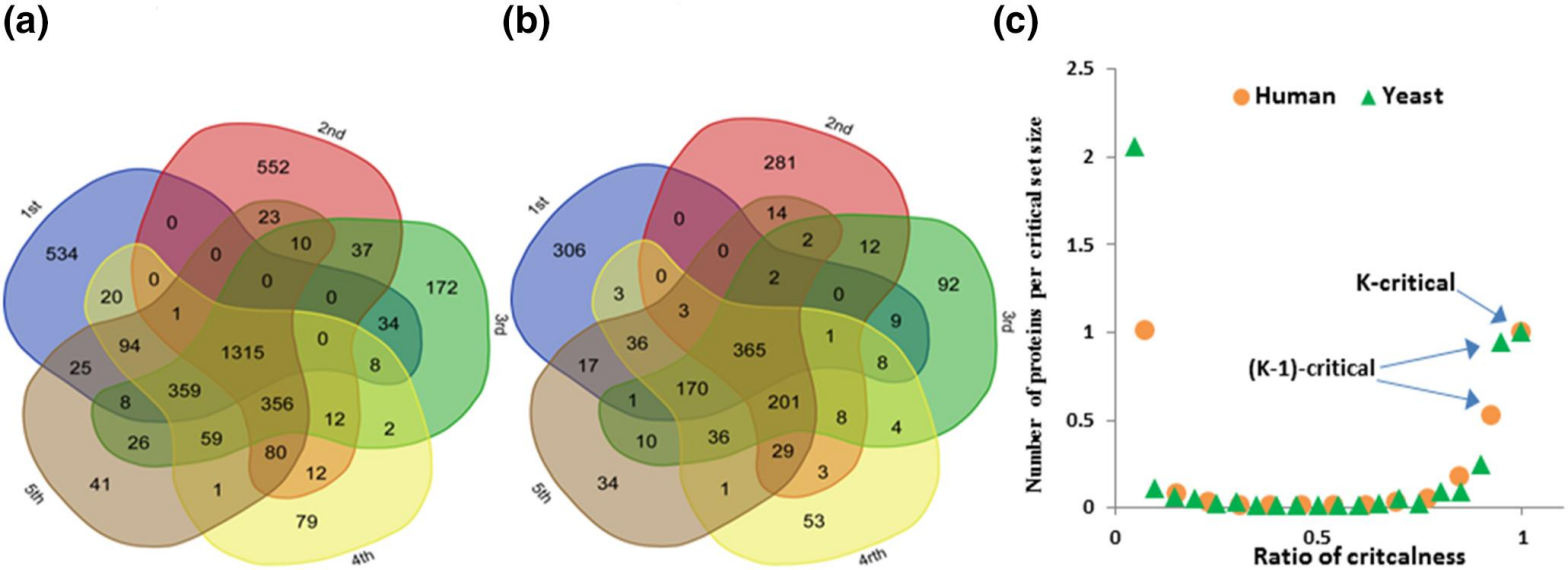
The multiple MDSets in HHQBP and YHQBP PPI networks using the ITR‐MDSets model. (a, b) Venn diagrams that show the overlap between the first five MDSets of each data set. (c) Bathtub curve that represents the trend (size and criticalness degree) of the grouped critical sets extracted from the generated MDSets using MOIA. The tool in [46] was used to draw figures (a) and (b)

The ITR‐MDSets model returns *k* MDSets (13 MDSets for the HHQBP data set and 20 MDSets for the YHQBP network). The critical set is the intersection among all these k MDSets, so the critical set was defined as the k‐critical set. Figure 6(a) shows the first five of 13 generated MDSets in the HHQBP data set, and Figure 6(b) shows the first five of 20 MDSets in the YHQBP data set (Table 6). Moreover, the proteins were grouped depending on their presence in the number of generated MDSets that they comprised. Then, these numbers were normalised using the size of the k‐critical set and the number of the generated MDSets. It was found that regardless of the PPI network, the same trend was obtained between the ratios of criticalness to the ratio of proteins that have the same criticalness as shown in Figure 6(c). This curve is like the bathtub curve that is used in the reliability theorem.

The critical set had a great interest in controllability [47]. Wuchty et al. [12] found that in PPI networks, the k‐critical proteins (kinase and transcription factors) play an important role in phosphorylation and regulatory events in their interactions. Despite this, important genes that do not present in the k‐critical set may be neglected, so the concept of criticalness was extended to other degrees of criticalness. Figures 6(a, b) show that there is a set of proteins that appears in (k−1) MDSets for each data set. Due to the large number of EGs in this set compared to the k‐critical set, this set is called the (k−1)‐critical set. Table 6 shows some MDSets generated by the ITR‐MDSets and the URD‐MDSets models for the HHQBP and YHQBP data sets along with the number of EGs in k‐ and (k−1)‐critical sets. To show the biological function of the (k−1)‐critical proteins, the number of EGs, KGs, and TFs proteins involved in the (k−1)‐critical set were counted (Table 8). It was found that the (k−1)‐critical set is as important as the k‐critical set. Thus, the authors believe that the (k−1)‐critical set analysis is as important as the critical set analysis and can be used for other networks or graph types.

**TABLE 8:** Comparison between k‐ and (k–1)‐critical sets extracted from HHQBP data set in the number of EGs [41], KGs [40], TFs [43], DGs [45], PHGs [45], and HKGs [9] (The Human Protein Atlas database) and comparison between k and (k–1)‐critical sets extracted from YHQBP data set in the EGs [41], KGs [42], and TFs [44]

	Critical set	No. of proteins	EGs	KGs	TFs	DGs	PHGs	HKGs
HHQBP	k‐Critical	1315	279	61	106	294	97	695
	(k−1)‐Critical	695	166	33	73	115	45	405
YHQBP	k‐Critical	364	93	9	9	‐	‐	‐
	(k−1)‐Critical	344	82	9	13	‐	‐	‐

*Abbreviations*: DGs, Drug‐target genes; EGs, Essential genes; HHQBP, high‐quality binary protein; HKGs, Housekeeping genes; KGs, Kinase genes; PHGs, pharmaceutics genes; TFs, Transaction Factors; YHQBP, high‐quality co‐complex protein.

### 5.7 Comparison with community detection methods

**FIGURE 7:**
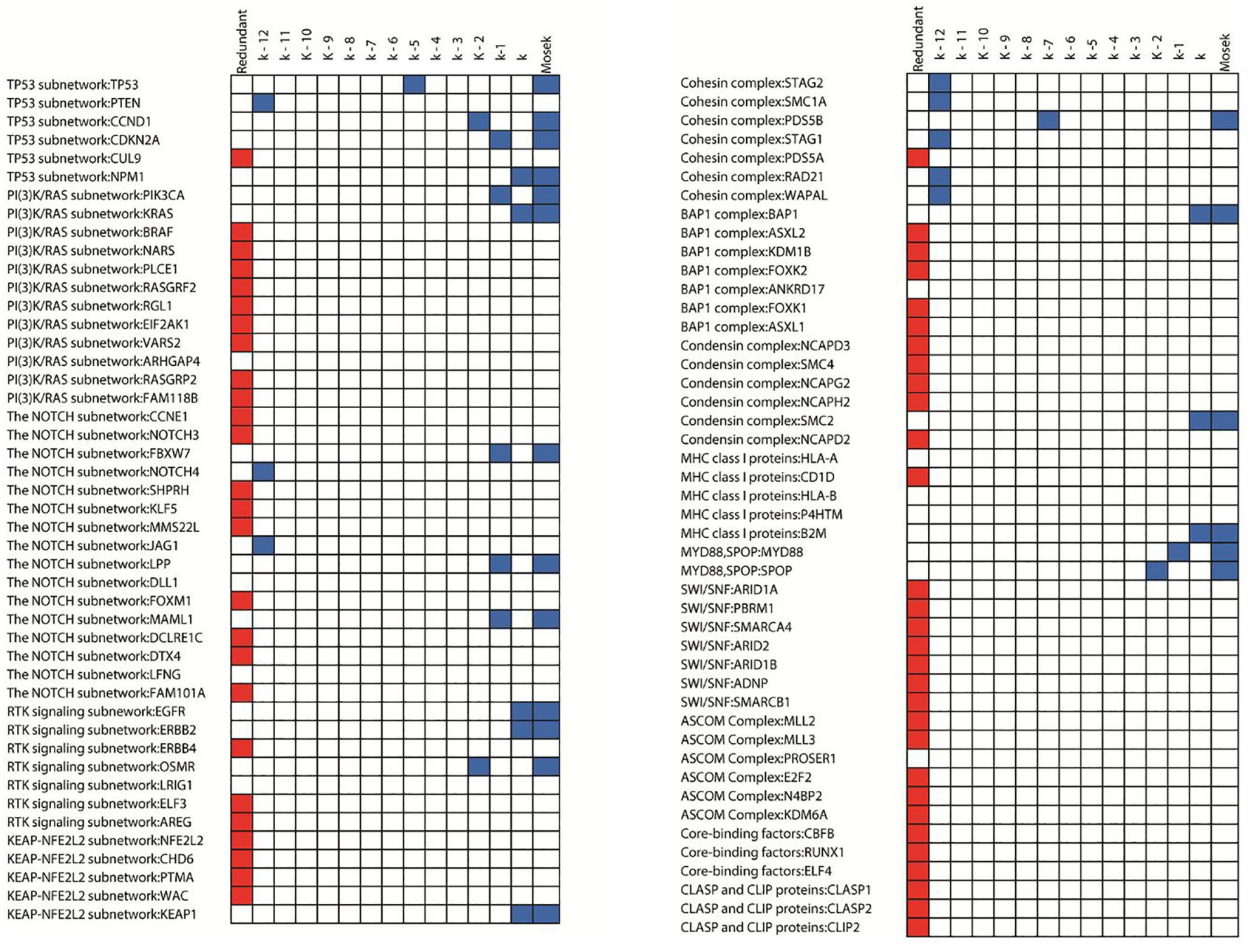
Comparison of detected subnetworks from HotNet2 and our critical sets. The blue square means that the protein is present in one or more of the MDSets. The red square means that the protein is in the redundant set, that is, it is not present in any MDSet

To validate types of proteins in the generated critical sets, they were compared with subnetworks extracted by the HotNet2 Algorithm [51]. HotNet2 integrated the PPI network with mutation information for 11,500 proteins in 12 cancer types from the TCGA project. The authors identified and annotated 15 significantly mutated subnetworks (Supplementary Table 5 in HotNet2 [51]). Figure 7 shows a comparison of the reported sets with HotNet2 subnetworks proteins and the basic model solution in Equation (1) using the Mosek solver, in the last column. It is noticeable that each protein present in the Mosek solution exists in one of the authors' critical sets. Nevertheless, several proteins appeared in the authors' critical sets but not in the Mosek solution. Additionally, some proteins in the subnetworks are not in existence at the HHQBP PPI network.

Each subnetwork in HotNet2 should contain one or more proteins that can dominate the other proteins in the subnetwork. The TP53 subnetwork has the highest covering score of 68% in HotNet2 and contains 45 subunits (Supplementary Table 8 in HotNet2 [51]). There are 18 proteins from this subnetwork in the authors' critical set and there are 23 proteins in the redundant set (the first column in red). Moreover, the authors found five proteins in the (k−1)‐critical set and four proteins in the k‐critical set. PTEN protein (the second mutated protein in TP53 subnetwork) was reported in the (k−12)‐critical set, which means that this protein is present in a small number of MDSets. Moreover, PTEN protein was not found in the MDSet generated by Mosek. The second important subnetwork is the PI3K/RAS subnetwork (with a covering score of 20%), where PIK3CA and KRAS proteins were present in the k‐ and (k−1)‐critical sets. The third subnetwork is the NOTCH1 subnetwork (with a covering score of 33%), where three proteins were present in the (k−1)‐critical set, and two proteins were present in the (k−12)‐critical set. Additionally, it was found that the Cohesin complex subnetwork has many proteins in the (k−12)‐ and (k−13)‐critical sets. Finally, the BAP1, condensing and MHC class I subnetworks are dominated by proteins in the k‐critical set.

From the covering concept of MDSets, proteins in the redundant set mean that these proteins do not belong to any generated MDSets. Figure 7 shows that the SWI/SNF and ASCOM subnetworks have only proteins in the redundant set, while no proteins in the authors' MDSets can dominate these complexes. Therefore, the authors extended their search to discover which proteins can dominate these complexes. They found that the protein SMARCD1 dominates the SWI/SNF complex, where the SMARCD1 protein was reported as a subunit in the SWI/SNF subnetwork. Additionally, they found that the protein NCOA6 dominates the ASCOM complexes. The protein NCOA6 has been reported to bind to the ASCOM complex [51]. Both SMARCD1 and NCOA6 proteins were present in the authors' critical sets and were not reported in the SWI/SNF and ASCOM subnetworks. Finally, for core‐binding factors for CLASP and CLIP proteins, it was found that each protein is connected to one or more proteins in the authors' critical sets.

To the best of the authors' knowledge, the MDSet was not used to predict the complex subunits or subnetworks from PPI network [52]. However, this analysis shows that the authors' MOIA method can be used with community detection methods to assist in annotating the predicted complexes.

## 6 CONCLUSIONS

Herein, the authors have introduced a new framework called MOIA, in which three models have been modified to generate multiple MDSets with minimum intersections for PPI networks. Using MOIA models, all PPI network nodes can be classified as critical, intermittent, and redundant nodes using a small number of iterations by the proposed algorithm. For example, the authors' models classified all nodes of the HHQBP data set using only 13 iterations (Table 4), however, traditional methods need thousands of iterations to classify these nodes [12, 20, 47]. Additionally, MOIA models allowed the authors to generate user‐defined MDSets with a maximum (or minimum) number of essential genes, protein kinases, and transcription factors. Moreover, the MOIA models were able to generate some MDSets that were not enriched with the essential genes. Thus, using the proposed models, the generated MDSet can be restricted, instead of increasing the number of nodes with specific features, such as the node degrees [13].

The authors also extended the concept of the nodes criticalness to identify (k−1),(k−2),…,1,0‐critical sets. It was found that the relationship between degrees of criticalness and protein ratios in each group follows the bathtub curve in reliability theory, regardless of the type of PPI network [Figure 6(c)]. The (k−1)‐critical set contains many essential genes, kinases, transcriptions factors, and drug targets, similar to the k‐critical set. Moreover, the (k−1)‐critical set represents a new analysis of PPI networks and can be used to predict new drug targets to be integrated with community detection methods. Finally, the proposed MOIA models can be applied to other network types and other areas of network analyses.
